# Patient-Centered Clinical Decision Support for Recommended Screenings in French Primary Care: Protocol for a Multicenter Mixed Methods Feasibility Study

**DOI:** 10.2196/89922

**Published:** 2026-09-03

**Authors:** Pierre-Yves Meunier, Laurent Magaud, Romain Varnier, Emmie Goetz, Melinda Bekaddour, Ludivine Albert, Inès Khati, Ilhem Bensekka, Julie Haesebaert, Denis Maillet, Julien Péron, Laurent Letrilliart

**Affiliations:** 1 Collège Universitaire de Médecine Générale Université Claude Bernard Lyon 1 Lyon, Auvergne-Rhône-Alpes France; 2 RESHAPE (RESearch on HealthcAre PErformance) Université Claude Bernard Lyon 1, INSERM U1290 Lyon France; 3 Hospices Civils de Lyon Pôle de Santé Publique Service Recherche et Epidémiologie Clinique, F-69000 Lyon France; 4 Hospices Civils de Lyon Service d’oncologie médicale Centre Hospitalier Lyon Sud, F-69000 Lyon France

**Keywords:** primary health care, clinical decision support systems, screening, shared decision-making, prevention, feasibility study, mixed methods

## Abstract

**Background:**

Primary care professionals (PCPs) are responsible for identifying eligibility and supporting patient decisions for a wide range of recommended screenings. However, the number of recommendations (42 for adults in France), complex eligibility criteria, limited consultation time, and competing priorities hinder their systematic delivery. Clinical decision support systems (CDSSs) with patient decision aids may help structure prevention-focused consultations and support shared decision-making, but their feasibility and acceptability in routine primary care remain uncertain. As part of the Aide aux Dépistages Recommandés (ADER) research program, we developed Lianeli, a CDSS designed to provide individualized, patient-centered support for all nationally recommended screenings.

**Objective:**

ADER-Faisabilité (ADER-F) is a study with the primary objective of assessing the feasibility of implementing Lianeli in routine primary care by estimating the proportion of patients for whom the CDSS is used to completion. Secondary objectives are to (1) quantitatively and qualitatively describe implementation feasibility, (2) describe organizational adjustments and interprofessional work related to the intervention, (3) explore the association between interprofessional use of Lianeli, health literacy, social deprivation, and feasibility, and (4 and 5) estimate the proportion of eligible, overdue or never screened patients who engage in organized or opportunistic cancer screening, respectively.

**Methods:**

ADER-F is a noncomparative, multicenter feasibility study with a mixed methods design conducted in primary care centers in the Auvergne-Rhône-Alpes region in France. We plan to recruit 210 adult patients from 21 general practitioners (GPs) across 17 sites. During routine consultations, eligible patients will be invited to complete a self-administered digital health questionnaire in Lianeli, which applies guideline-based algorithms to identify recommended screenings. Within 60 days, patients will attend a dedicated screening consultation during which the GP will validate the patient’s responses, discuss benefits and harms of each screening using shared decision-making, and update the patient’s screening pathway. Quantitative data will include Lianeli metadata, sociodemographic characteristics, health literacy, social deprivation, implementation feasibility measures (Guideline Implementation with Decision Support [GUIDES]–based questionnaires), and usability measures (French version of the System Usability Scale [F-SUS]). Semistructured interviews with patients and PCPs will be analyzed thematically and integrated with quantitative findings in a convergent metasynthesis.

**Results:**

The ADER-F study was funded in 2023. Following ethics approval in October 2025 and French National Commission on Data Protection authorization in February 2026, recruitment started in March 2026, with a planned 2-month inclusion period and an overall study duration of 6 months. The results are expected to be published in 2027.

**Conclusions:**

ADER-F will provide evidence on the feasibility and acceptability of a patient-centered CDSS to support recommended screenings in French primary care. The findings will identify determinants of successful implementation, guide possible developments of the Lianeli software and care pathways, and determine whether a subsequent effectiveness study is justified.

**Trial Registration:**

ClinicalTrials.gov NCT07270926; https://clinicaltrials.gov/study/NCT07270926

**International Registered Report Identifier (IRRID):**

PRR1-10.2196/89922

## Introduction

### Background

Primary care professionals (PCPs), particularly general practitioners (GPs), play a central role in identifying patients eligible for recommended screenings, informing them of potential benefits and harms, and supporting shared decision-making [1,2]. In France, the French National Authority for Health (Haute Autorité de Santé; HAS) produces recommendations for organized screenings (eg, breast, colorectal, and cervical cancer) and opportunistic screenings (eg, hypertension), as well as other preventive interventions (eg, behavioral counseling about tobacco). The number of these recommendations (n=42 for adults) and eligibility criteria make it challenging to systematically identify all eligible patients in everyday practice and to properly inform patients about these screenings [3].

In addition to this complexity, several barriers limit the delivery of preventive care, including screenings, in primary care settings [4]. The lack of time during consultations and the need to prioritize acute or chronic issues over prevention are frequently reported by GPs [5,6]. In France, the mean GP consultation time was 17 minutes in 2014 [7]. Patients who consult infrequently are particularly at risk of missing opportunities for prevention [8]. Patients also lack information on screening options, which can impede informed decisions [9]. Inadequate health literacy and social deprivation are additional sources of inequity in the uptake of recommended screenings [10,11].

Clinical decision support systems (CDSSs) are designed to provide patient-specific assessments or recommendations to support clinical decision-making [12]. For prevention and screening, they could match patient characteristics with guideline-based algorithms to identify eligibility, communicate benefits and risks with patient decision aids, and help structure shared decision-making discussions [13]. CDSSs can improve PCPs’ adherence to guidelines [14]. Patient decision aids may reduce decisional conflict and support self-efficacy [15]. According to PCPs, the successful implementation of CDSSs in real-world settings depends on usability, integration into workflows, and alignment with professional expectations [16].

The Aide aux Dépistages Recommandés (ADER) research program supports the design, implementation, and evaluation of a complex intervention centered on the use of a patient-centered CDSS called Lianeli [17]. Lianeli uses a self-administered health questionnaire to collect individual information (eligibility criteria) and then applies guideline-based algorithms from the HAS national guidelines to identify eligibility for recommended screenings. The tool generates tailored information about screening eligibility, including explanations of risk stratification, potential benefits and harms, and provides a summary that can be integrated into the electronic health record (EHR). This information can be discussed during a dedicated screening consultation. Such a dedicated consultation was introduced in 2024 by the French National Health Insurance Fund to promote preventive care [18].

### Study Objectives

The primary objective of the ADER-Faisabilité (ADER-F) study is to evaluate the feasibility of implementing the Lianeli-based intervention in primary care by measuring the proportion of participants for whom the Lianeli CDSS is used to completion.

Secondary objective 1 is to describe, quantitatively and qualitatively, the feasibility of implementing the intervention according to the 4 domains of the Guideline Implementation with Decision Support (GUIDES) checklist. Secondary objective 2 is to qualitatively describe organizational adjustments and interprofessional collaboration within participating sites. Secondary objective 3 is to explore whether interprofessional use of Lianeli, patient health literacy, and social deprivation are associated with complete use of Lianeli (the primary outcome). Additional secondary objectives are to describe, among participants who are overdue or have never been screened, the proportion who engage in the screening pathway, considering separately participants eligible for organized cancer screenings, including breast, colorectal, and cervical cancer (secondary objective 4), and those eligible for opportunistic cancer screenings (secondary objective 4).

## Methods

### Study Design

**Figure 1:**
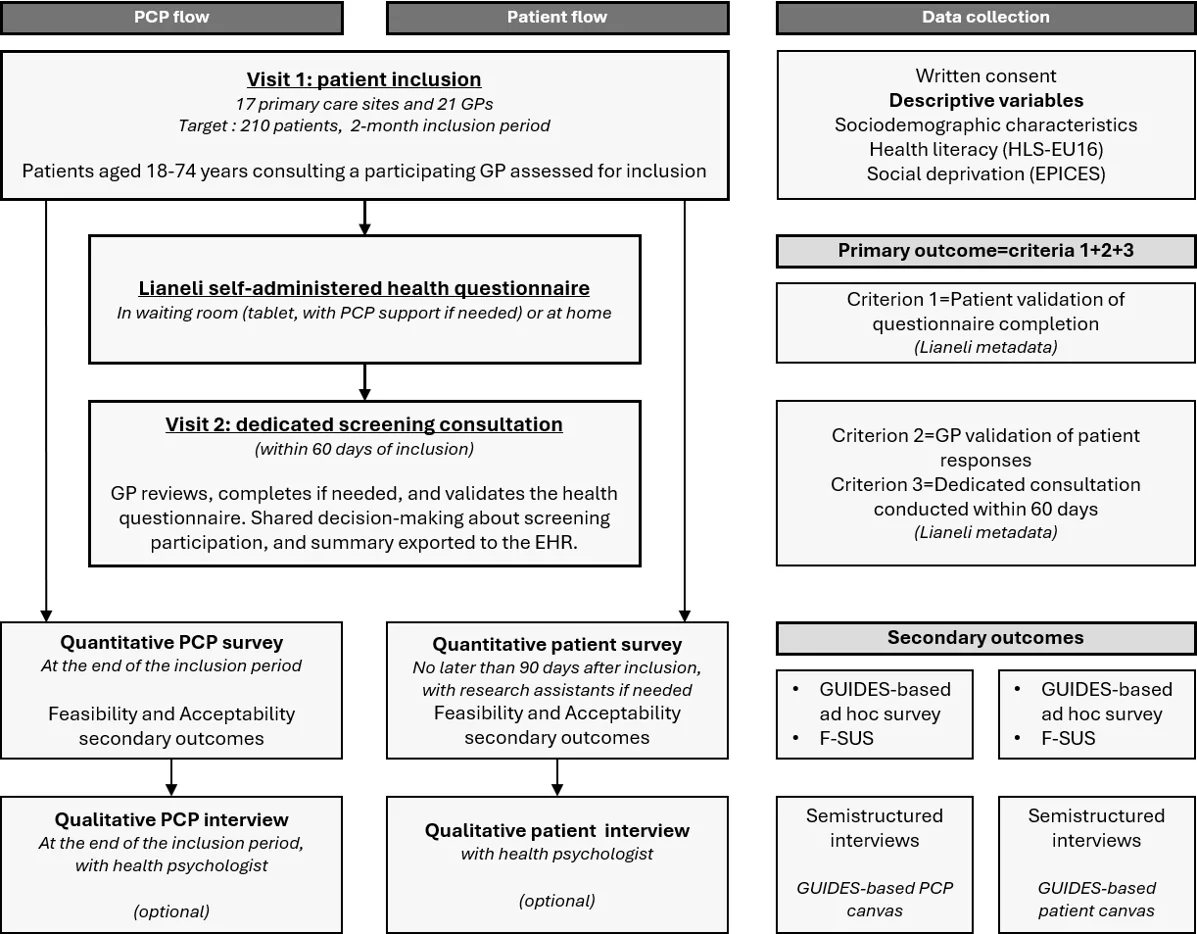
Design of the Aide aux Dépistages Recommandés-Faisabilité (ADER-F) study: participant flow and corresponding data collection. EHR: electronic health record; EPICES: Evaluation of Precarity and Inequalities in Health Examination Centres; F-SUS: French version of the System Usability Scale; GP: general practitioner; GUIDES: Guideline Implementation with Decision Support; HLS-EU16: European Health Literacy Survey Questionnaire-16; PCP: primary care professional.

ADER-F is a prospective, regional, multicenter, noncomparative feasibility study conducted in routine primary care. It corresponds to step 2 in the development and evaluation of a complex health intervention [19]. The study applies mixed methods to integrate quantitative and qualitative data in a convergent metasynthesis [20]. The study draws on the GUIDES theoretical framework, a reference for assessing determinants of successful implementation of guideline-based CDSSs [21]. This framework comprises 16 criteria, evenly distributed across 4 domains: enabling context, appropriate content, effective system, and effective implementation. Textbox 1 presents key characteristics of the study design. The full description of GUIDES indicators evaluated in the present study is available in Multimedia Appendix 1. Figure 1 presents the design of the study, including the anticipated participant flow and the data collected at each step.

**Key characteristics of the ADER-F (Aide aux Dépistages Recommandés-Faisabilité) feasibility study.**

**Study design**

Noncomparative, multicenter, regional feasibility study with a mixed methods design

**Study setting**

Primary care practices in the Auvergne-Rhône-Alpes region, France

**Population**

Patients aged 18-74 years consulting participating general practitioners (GPs)

**Intervention**

Use of the Lianeli clinical decision support system (CDSS) and a dedicated screening consultation

**Primary objective**

To estimate the proportion of patients for whom Lianeli is used completely in routine care

**Secondary objectives**

Implementation feasibility (Guideline Implementation with Decision Support [GUIDES] domains), organizational impact, effect of interprofessional use, health literacy, and social deprivation on the primary outcome, and potential impact on screening participation

**Planned sample size**

21 GPs and at least 210 patients (≈10 patients per GP)

**Planned study duration**

6 months (2-month inclusion period plus follow-up and qualitative interviews)

**Primary outcome**

Proportion of patients with complete use of Lianeli (patient questionnaire completed, GP validation of the questionnaire, and screening consultation within 60 days)

**Data collection**

Lianeli metadata, patient and primary care professional (PCP) questionnaires, semistructured interviews

**Analysis**

Descriptive and exploratory quantitative analyses, thematic qualitative analysis, and convergent mixed methods metasynthesis guided by the GUIDES framework

This protocol is reported in accordance with the Consensus Reporting Items for Studies in Primary Care (CRISP) 2023 statement [22]. As an interventional study, this protocol is reported in accordance with the applicable items of the SPIRIT (Standard Protocol Items: Recommendations for Interventional Trials) 2013 statement: the completed checklist is provided in Multimedia Appendix 2 [23]. The mixed methods components will be reported following the Good Reporting of a Mixed Methods Study framework, and the qualitative components will follow the Consolidated Criteria for Reporting Qualitative Research (COREQ) [24,25].

### Setting

The study will be conducted in 17 primary care centers (participating sites) involving 21 GPs in the Auvergne-Rhône-Alpes region of France. Participating sites will include solo GP practices, one community health care center, and multiprofessional group practices. They may involve other PCPs in the delivery of the intervention (team-based practices), depending on the local organization, as long as a GP conducts both the inclusion and dedicated screening consultations. Before patient recruitment, all participating GPs will receive a standardized online training session on the use of the Lianeli CDSS and on the study procedures, delivered by the research team. There will be no restriction on concomitant care during the study.

### Eligibility Criteria

Table 1 describes the patient and PCP eligibility criteria.

**Table 1:** Study eligibility criteria.

Population and category			Criteria
**Patients**			
	Inclusion	Age 18-74 years Consulting a participating GP^a^ in the Auvergne-Rhône–Alpes region Covered by the French national health insurance Able to understand and write in French sufficiently to complete the questionnaire (with PCP^b^ support if needed) Having provided written informed consent to participate in the study	
	Noninclusion	Pregnant or breastfeeding woman Under legal protection Presenting with a psychiatric disorder or psychiatric symptoms associated with impaired judgment, as assessed by the investigator Participating in another clinical study that could interfere with the ADER-F^c^ study	
	Exclusion	Withdrawal of consent	
**GPs**			
	Inclusion	GPs working in primary care settings in the Auvergne-Rhône-Alpes region	
**Other PCPs**			
	Inclusion	Health professionals (eg, nurses and medical assistants) working in participating sites	

^a^GP: general practitioner.

^b^PCP: primary care professional.

^c^ADER-F: Aide aux Dépistages Recommandés-Faisabilité.

### Recruitment and Informed Consent

#### PCPs and Patient Recruitment

We recruited PCPs through university-affiliated teaching practices via email.

Patients will be recruited during routine consultations, as part of usual care, with participating GPs (visit 1). The GP will present the study and verify eligibility criteria. Patients who agree to participate will receive oral and written information describing the objectives, procedures, potential benefits and risks, data confidentiality, and their rights (including the right to withdraw at any time without consequences for their care). Written informed consent will be obtained before any data collection specific to the research.

#### Recruitment for Qualitative Interviews

Qualitative semistructured interviews will be conducted by a health psychologist with a purposive sample of volunteer patients and PCPs to assess the feasibility of implementing the Lianeli system. Their agreement to participate in the interviews will be documented in the written consent form. Participation in these interviews is contingent upon obtaining written authorization for audio recording and the use of the recorded data. Nonsampled patients or PCPs will be notified by email or mail. We will aim for maximum variation in sex, age, and health literacy for patients, and in sex, age, and diversity in roles for PCPs. Up to 24 interviews per group are planned, with early termination possible upon data saturation. Among PCPs (GPs and other professionals), semistructured interviews will be performed at the end of the patient recruitment phase in each center.

### The Lianeli CDSS

Lianeli is a CDSS that provides individualized assessment of eligibility for recommended screenings based on the French HAS guidelines. The system is designed to collect relevant patient information (eg, age, sex, personal and family history, and risk behaviors) via a self-administered digital health questionnaire, and to automatically apply the French HAS national screening recommendations to this information to determine which screenings are recommended for each patient. To support shared decision-making during a dedicated screening consultation, Lianeli then provides factual, patient-friendly information on the practical considerations of each recommended screening and, when available, on the benefits and harms of screening options. Finally, the system generates a structured summary of screening eligibility and decision outcomes, designed for inclusion in the patient’s EHR at the end of the consultation. As Lianeli currently operates as a standalone web-based application, transfer to the EHR requires a manual step.

The Lianeli CDSS was developed by a scientific interdisciplinary committee from a systematic review of French screening recommendations [3] and then tested internally against clinical vignettes and real cases for reliability. The system adopts a patient-centered approach, empowering patients to complete the questionnaire independently (or with assistance) and equipping them with the information needed to prepare for the discussion with their GP. This integrated approach distinguishes Lianeli from existing tools, which often focus on a single pathology or do not support shared decision-making.

### Timeline

The ADER-F study is structured into 4 main steps (Figure 1). At least 210 patients will be included, and 21 GPs will be recruited over a 2-month period, with a total study duration of 6 months. The expected participation duration is between 1 day and 3 months for patients (extended by up to 1 additional month for those participating in qualitative interviews) and 6 months for PCPs.

#### Step 1 (Visit 1): Inclusion Consultation (Day 1)

The GP identifies an eligible patient, presents the study, and obtains informed written consent. A time for reflection will be systematically proposed. After visit 1, the patient is invited to complete the self-administered digital health questionnaire within the Lianeli, either in the waiting room with a tablet computer, with assistance from a PCP if needed (according to local organization), or at home within 28 days.

#### Step 2 (Visit 2): Dedicated Screening Consultation (Day 1 to Day 60)

Within 60 days of inclusion, the patient attends a dedicated consultation focused on screening. The GP reviews, completes, if needed, and validates the health questionnaire within Lianeli. The GP engages in a discussion with the patient regarding the eligibility and personal relevance of recommended screenings, weighing benefits against potential harms. A summary of screening eligibility and shared decision-making outcomes is integrated into the EHR.

#### Step 3: Quantitative Survey

After visit 2, patients complete a survey based on the GUIDES patient checklist (Multimedia Appendix 3). After achieving their inclusion targets, PCPs complete a similar GUIDES-based implementation survey (Multimedia Appendix 4). These GUIDES-based questionnaires combine validated instruments (French version of the System Usability Scale [F-SUS]) with items constructed for the study and have not undergone psychometric validation [26].

#### Step 4: Qualitative Interviews

Patient interviews will occur within 1 month after study completion (early withdrawal included), and PCP interviews within 1 month after the last patient’s last visit at each site.

These interviews explore different dimensions of feasibility and usability: perceived relevance and acceptability of the intervention, perceived impact on workloads and workflow, quality and timing of information, interprofessional collaboration, and ethical and data security concerns (Multimedia Appendix 5).

### Outcomes

#### Overview

In the ADER-F study, feasibility refers to the extent to which the intervention can be delivered as intended in routine primary care; it is assessed primarily through the proportion of patients with complete use of Lianeli (primary outcome) and through objective CDSS use and data quality metrics (Table 2). Acceptability refers to the extent to which patients and PCPs consider the intervention appropriate, satisfactory, and usable; it is assessed through the GUIDES-based surveys, the F-SUS usability score, and the qualitative interviews.

#### Primary Outcome

The primary outcome is the proportion of included patients for whom the Lianeli CDSS is used completely. Complete use is defined as meeting all 3 of the following criteria: the patient validates completion of the Lianeli self-administered health questionnaire (criterion 1), the GP checks data quality and validates the patient’s responses (criterion 2), and the dedicated screening consultation is conducted within 60 days after the inclusion consultation (criterion 3). This outcome is binary at the patient level (complete vs incomplete use).

#### Secondary Outcomes

Secondary outcomes are grouped according to study objectives (Table 2).

**Table 2:** Study objectives (SOs) and secondary outcomes: data sources, timing of measurement, and planned analyses.

SOs and secondary outcomes		Data source	Timing of measurement	Planned analysis
**SO1: quantitative feasibility criteria**				
	Percentage of Lianeli health questionnaires started and completed, percentage validated by GPs^a^, number of dedicated consultations, missing data rates before vs after GP review of the health questionnaire, percentage of documented previous screening tests, and patient error rate regarding past examinations	Lianeli CDSS^b^ (automatically recorded metadata)	Continuously recorded from visit 1 to visit 2 (day 1 to day 60)	Descriptive statistics
	GUIDES^c^ patient survey (22 items, including the F-SUS^d^; Multimedia Appendix 3)	eCRF^e^ or paper-based survey, with the help of research assistants if needed	After visit 2, no later than 90 days after inclusion	Descriptive statistics
	GUIDES PCP^f^ survey (22 items, including the F-SUS) (Multimedia Appendix 4)	eCRF or paper-based survey	At the end of the inclusion period	Descriptive statistics
**SO1: qualitative feasibility criteria**				
GUIDES-related interview canvas (Multimedia Appendix 5)		Semistructured interviews	Patients: within 1 month after study completion; PCPs: within 1 month after the last patient’s last visit at each site	Thematic analysis with deductive (GUIDES domains) and inductive coding; NVivo software
**SO2: organizational adjustment and interprofessional collaboration**				
How centers adapt their organization to implement the Lianeli-based intervention (integration into preventive programs and workflows; patterns of interprofessional collaboration		Semistructured interviews	Patients: within 1 month after study completion; PCPs: within 1 month after the last patient’s last visit at each site	Descriptive qualitative synthesis; organizational model derived from qualitative findings
**SO3: explanatory variables for feasibility**				
	Interprofessional use of Lianeli, defined as the involvement of ≥2 health professionals (including the GP) working with the patient during ADER-F	eCRF by research assistants	Documented during the intervention period (day 1 to day 60)	Exploratory regression model on the primary outcome
	Patient health literacy (HLS-EU16^g^, French version; range 0-16; inadequate ≤8, problematic >8 and ≤12, adequate >12)	eCRF or paper-based survey, with the help of research assistants if needed	At inclusion (visit 1)	Descriptive statistics
	Social deprivation (EPICES^h^ score; range 0-100; higher=more deprived; threshold of 30.2 commonly used)	eCRF or paper-based survey, with the help of research assistants if needed	At inclusion (visit 1)	Descriptive statistics
**SO4 and SO5: exploratory signals related to cancer screening participation**				
Among patients eligible for at least 1 organized cancer screening (breast, colorectal, or cervical cancer) who are overdue or have never been screened: proportion attending the dedicated screening consultation within 60 d of inclusion; the same proportion is described among patients eligible for opportunistic cancer screenings		Lianeli CDSS (automatically recorded metadata)	Within 60 days of inclusion	Descriptive statistics (hypothesis-generating)

^a^GP: general practitioner.

^b^CDSS: clinical decision support system.

^c^GUIDES: Guideline Implementation with Decision Support.

^d^F-SUS: French version of the System Usability Scale.

^e^eCRF: electronic case report form.

^f^PCP: primary care professional.

^g^HLS-EU16: European Health Literacy Survey Questionnaire-16.

^h^EPICES: Evaluation of Precarity and Inequalities in Health Examination Centres [Evaluation de la Précarité et des Inégalités de santé dans les Centres d'Examens de Santé].

### Sample Size

As a feasibility study, ADER-F is not designed to detect clinical effects on practice or patient outcomes but to estimate feasibility and acceptability parameters. No formal hypothesis-testing power calculation was performed. Instead, a minimum sample size of 210 patients (approximately 10 patients per GP, across 21 GPs) was chosen to allow precise estimation of the primary feasibility outcome.

A sample size of 210 patients ensures that the SE of the estimated proportion of complete Lianeli use remains below 4%, irrespective of the observed rate. Consequently, observing a complete use rate of at least 77% will allow us to conclude that the true participation rate exceeds 70% (with a 2-sided type I error of 5%).

### Data Collection and Management

All quantitative data will be recorded in an electronic case report form (eCRF) hosted on a secure platform. To promote participant retention and complete follow-up, research assistants will contact each patient by telephone within 7 working days of inclusion to collect baseline data and help with Lianeli connection if needed. Research assistants will offer telephone completion of the GUIDES questionnaires to patients and PCPs who have not responded within 14 days.

A health psychologist trained in qualitative methods will conduct semistructured individual interviews with patients and individual interviews with PCPs. These sessions will take place within 1 month of the last patient visit, or for PCPs, within 1 month of the last patient visit at each site.

Interviews will be guided by a predefined thematic canvas derived from the GUIDES checklist and iteratively enriched with emerging themes identified during data collection. Interviews will be audio-recorded, transcribed verbatim, anonymized, and imported into qualitative analysis software within 1 month of recording. Audio files will be destroyed after transcription and quality checking.

### Statistical Analysis

A data monitoring committee will validate, before statistical analyses, the patient sample to be analyzed, in accordance with the protocol. No interim analyses have been planned. The primary analysis population will be the intention-to-treat population, defined as all included patients regardless of eligibility deviations or level of participation in the intervention. Patients who withdraw or are lost to follow-up will not be replaced.

Quantitative variables will be summarized using standard descriptive statistics: number of observations, number of missing values, mean, SD, median, IQR, minimum, and maximum. When relevant, quantitative variables may be categorized using the median or thresholds based on the literature. Categorical variables will be described using frequencies and percentages (with missing values reported but excluded from denominators).

The proportion of patients with complete use of Lianeli will be estimated with its 95% CI and compared with a prespecified threshold of 70% using a 2-sided exact binomial test. A proportion statistically significantly greater than or equal to 70% will be considered evidence of feasibility. The primary outcome will be evaluated at the end of the study. Secondary quantitative outcomes (Lianeli metadata, questionnaire scores, and screening participation proxies) will be analyzed descriptively. Exploratory analyses will examine associations between the primary outcome and selected determinants of Lianeli use. Given the exploratory nature and the limited sample size of this feasibility study, the variables included in the regression models were prespecified based on clinical and methodological relevance: interprofessional use of Lianeli, health literacy (European Health Literacy Survey Questionnaire-16 [HLS-EU16] score) [27], and social deprivation (Evaluation of Precarity and Inequalities in Health Examination Centres [EPICES] score) [28]. Associations will be described using regression models and reported as effect estimates with their 95% CIs. These analyses will be considered exploratory and hypothesis-generating.

Missing data will be considered informative in this feasibility study. They will be described explicitly and taken into account in the analysis of outcomes, but no imputation or correction methods will be applied.

### Qualitative Analysis

Qualitative data will be analyzed using a thematic content analysis. The thematic analysis will follow the stages recommended by Braun and Clarke [29] (reading, coding, and categorization into themes and subthemes refined iteratively). It will be conducted using NVivo thematic analysis software (version 1.4; Lumivero). Transcripts will be coded with both deductive codes based on the GUIDES domains and inductive codes emerging from the data. Vertical (within-interview) and transversal (across interviews) analyses will be conducted to identify themes and subthemes.

Triangulation will be performed across data sources (patients vs PCPs) and across centers, with particular attention to interprofessional dynamics within multidisciplinary practices. The qualitative findings will then be integrated with quantitative results in a convergent mixed methods metasynthesis guided by the GUIDES framework, to provide a comprehensive understanding of feasibility and acceptability.

### Ethical Considerations

The ADER-F study protocol was approved by an ethics committee (Comité de Protection des Personnes Ouest III, decision 25.01942.000426#1) in October 2025. All included patients and PCPs provide written informed consent. A reflection period is systematically offered before consent is obtained, and no research-specific data are collected beforehand. Participants taking part in the qualitative interviews provide separate written authorization for audio recording and use of the recorded data. Model consent forms are available from the corresponding author on request. Substantial amendments will be submitted to the ethics committee and updated in the trial registry.

Regarding privacy and confidentiality, this study was authorized by the French National Commission for Data Protection (Commission Nationale de l’Informatique et des libertés; CNIL) in February 2026. All research data are pseudonymized at the point of collection and stored on a secure, access-controlled electronic database. Qualitative interview recordings are transcribed, anonymized, and destroyed after transcription and quality checking, as detailed in the Data Collection and Management section.

Patients participating in semistructured qualitative interviews receive financial compensation. Participating GPs receive compensation for their training on the CDSS, as well as for the additional time required by the study-specific procedures (inclusion, dedicated screening consultation, and participation in a qualitative interview).

## Results

The ADER-F study was funded by the Cancéropôle Lyon Auvergne-Rhône-Alpes (CLARA), the Métropole de Lyon, and the Auvergne-Rhône-Alpes region under the Proof of Concept 2023 program. The protocol received ethics approval from the Comité de Protection des Personnes Ouest III in 2025 (25.01942.000426#1) and was registered on ClinicalTrials.gov (NCT07270926). Recruitment began in March 2026, with a planned 2-month inclusion period and a total study duration of 6 months. As of manuscript submission, no participant had been enrolled, and data collection had not started. Data collection and analysis are expected to be completed in July 2026 and by the end of 2026, respectively. The publication of results is anticipated in 2027.

## Discussion

### Comparison With Other Screening CDSSs

Most screening CDSSs address only one or a limited number of primary care screenings. Among these tools, the most extensively studied is a United States–based system developed to screen for 8 pediatric conditions [30]. In France, Lianeli is the first CDSS to cover all screenings recommended by the HAS and to provide decision support aids.

### Implications

The ADER-F study is expected to provide a comprehensive assessment of the feasibility of a patient-centered and guideline-based CDSS (Lianeli) supporting recommended screenings in French primary care. The quantitative and qualitative indicators collected under the GUIDES framework will identify which contextual, content-related, system-related, and implementation factors facilitate or hinder the successful use of the CDSS. This step is essential to consider the large-scale deployment of the CDSS in primary care clinical practice.

The study will also explore how health literacy, social deprivation, and interprofessional collaboration influence feasibility. This is particularly important given known inequalities in access to digital services [31] and the complexity of organizing structured screening consultations in busy primary care practices.

If ADER-F demonstrates that the Lianeli-based intervention is feasible, the results will guide future developments of the software and adaptations to the organizational model (including interprofessional collaboration) before designing an evaluation of effectiveness. Positive feasibility findings could justify a randomized trial to assess its impact on screening participation and shared decision-making.

### Strengths and Weaknesses

Data collection was designed to have minimal impact on PCPs’ workflow, thereby preserving external validity. This study relies on a mixed methods and interdisciplinary approach, integrating complementary perspectives and enabling triangulation of findings. Moreover, the study is grounded in a validated conceptual framework, providing a robust theoretical basis to guide design and analysis and enhancing comparability with prior work.

Several limitations should be acknowledged. The primary outcome may be influenced by study-specific incentives that may not persist under real-world implementation conditions, including PCP compensation, support from research assistants, and a potential Hawthorne effect. In addition, the decision-aid library within the CDSS will not be fully available to patients and PCPs for each screening, as it is being developed in parallel. Although the evaluation protocol encompasses multiple outcomes across multiple domains, participant burden was deliberately minimized: primary outcome data are collected automatically through CDSS log data, additional questionnaires are administered with the support of research assistants, and qualitative participation remains optional. Finally, participating patients and PCPs are volunteers and may differ from the broader population in their engagement with preventive care. This self-selection bias may limit the generalizability of the feasibility estimates, and it will be considered when interpreting the findings and planning subsequent studies.

### Conclusions

The ADER-F feasibility study will assess whether a patient-centered CDSS for recommended screenings, implemented through dedicated screening consultations in primary care, can be integrated into routine practice and accepted by patients and primary care practitioners. By combining detailed implementation indicators with qualitative insights grounded in the GUIDES framework, the study will provide critical information for refining the intervention and determining whether a subsequent effectiveness trial is warranted. Ultimately, the Lianeli CDSS aims to support more systematic and patient-centered delivery of screenings in primary care.

## Appendix

Multimedia Appendix 1 Guideline Implementation with Decision Support (GUIDES) indicators evaluated in ADER-F (Aide aux Dépistages Recommandés-Faisabilité).

Multimedia Appendix 2 SPIRIT 2013 checklist.

Multimedia Appendix 3 Patient Guideline Implementation with Decision Support (GUIDES) questionnaire.

Multimedia Appendix 4 Primary care professional (PCP) Guideline Implementation with Decision Support (GUIDES) questionnaire.

Multimedia Appendix 5 Predefined qualitative canvas, according to the Guideline Implementation with Decision Support (GUIDES) framework.

## Abbreviations

ADER: Aide aux Dépistages Recommandés
ADER-F: Aide aux Dépistages Recommandés-Faisabilité
CDSS: clinical decision support system
CNIL: Commission Nationale de l’Informatique et des libertés
COREQ: Consolidated Criteria for Reporting Qualitative Research
CRISP: Consensus Reporting Items for Studies in Primary Care
eCRF: electronic case report form
EHR: electronic health record
EPICES: Evaluation of Precarity and Inequalities in Health Examination Centres
F-SUS: French version of the System Usability Scale
GP: general practitioner
GUIDES: Guideline Implementation with Decision Support
HAS: Haute Autorité de Santé
HLS-EU16: European Health Literacy Survey Questionnaire-16
PCP: primary care professional
SPIRIT: Standard Protocol Items: Recommendations for Interventional Trials

## Data Availability

All data will be analyzed after completion of the study and database lock. Deidentified datasets and the statistical analysis plan will be available from the corresponding author upon reasonable request, in accordance with French data protection regulations and the commitments made to participants. Study results will be disseminated through peer-reviewed publications and presentations at scientific conferences. Summaries of key findings will also be shared with participating general practitioners (GPs) and, where feasible, with patient participants.
