# Supplementary material for: Patient-Centered Clinical Decision Support for Recommended Screenings in French Primary Care: Protocol for a Multicenter Mixed Methods Feasibility Study
Source: JMIR Res Protoc. 2026 Sep 3;15:e89922. doi: 10.2196/89922 (PMC13586705; doi:10.2196/89922)
Supplement: Supplementary file 1 [file resprot_v15i1e89922_app1.docx]

**Multimedia Appendix 1**: Guideline Implementation with Decision Support (GUIDES) indicators evaluated in ADER-F (*Aide aux Dépistages Recommandés-Faisabilité*).

| GUIDES Checklist | | **Indicator** | | **Evaluated in ADER-F study** |
| --- | --- | --- | --- | --- |
| Domain 1: The CDSS context | |  | |  |
| CDSS can achieve the planned quality objectives | | - Does the available evidence support the use of CDS for the given outcomes, tasks and settings? | | X |
|  |  | - Does CDS address the factors that explain the current behaviour of healthcare providers and patients? | | X |
| The quality of the patient data is sufficient | | - Is the structured patient data that is needed to achieve the CDS objective sufficiently accurate and complete to allow the use of CDS? | | X |
|  |  | - If necessary, can the quality of the data be improved or can the CDS itself improve the data quality? | | X |
| Stakeholders and users accept CDSS | | - Do the users and stakeholders have a positive attitude towards the use of CDS? | | X |
|  |  | - Is there a clear benefit to the users who will engage with the CDS? | |  |
|  |  | - If necessary, is it possible to increase user and stakeholder acceptance? | | X |
| CDSS can be added to the existing workload | | - Is it feasible to introduce CDS, given the current workload and the usual work processes? | | X |
|  |  | - If necessary, can the workload or the work processes be changed or can the CDS system improve the workload or work processes? | | X |
|  |  | - Is the required hardware available and what will the impact be of adding CDS to the existing information systems? | | x |
| Domain 2: The CDSS content | |  | |  |
| The content provides trustworthy evidence-based information | | - Do the organization(s) and people that developed the decision support have credibility? | |  |
|  |  | - Is the advice supported by up-to-date scientific evidence and is the type and quality of this evidence clear to the user? | | X |
|  |  | - Is the decision support clear on the benefits and harms of the different management options? | | X |
| The decision support is relevant and accurate | | - Does the decision support address the information needs of the users? | | X |
|  |  | - Is it clear to the users why the decision support information is provided for a given patient? | | X |
|  |  | - Does the decision support contain accurate information that is pertinent to the care of the patient? | | X |
| The decision support provides an appropriate call to action | | - Is the recommended action clear enough for the targeted users to act on? | | X |
|  |  | - Is the clinical importance and urgency of the recommended action sufficiently clear? | | X |
|  |  | - Is the advice applicable in the setting in which it will be implemented? | |  |
|  |  | - Is it clear how the recommended action fits with other current guidelines? | |  |
| The amount of decision support is manageable for the target user | | - Is the amount of decision support per patient manageable for the health care provider? | | X |
| Domain 3: The CDSS system | |  | |  |
| The system is easy to use | | - Is it easy for users to interact with the CDS system? | | X |
|  |  | - Does the system facilitate (or, at least, not hinder) the workflow of the healthcare providers? | | X |
|  |  | - Can the system be customized to provide better user support | |  |
|  |  | - Is the system always up and running? | |  |
| The decision support is well delivered | | - Is the advice delivered in an appropriate mode, format and channel? | | X |
|  |  | - -s the display of the decision support eye-catching, intuitive, concise, consistent and unambiguous? | | X |
|  |  | - Is it appropriate to use specific functions (e.g. pop-ups, computerized restrictions, indications of (dis)agreement) for prioritized decision support? | | X |
| The system delivers the decision support to the right target person | | - Is the system able to facilitate team processes when these are needed? | | X |
|  |  | - Is the system reaching the targeted users (healthcare providers and/or patients)? | |  |
| The decision support is available at the right time | | - Does the system provide the decision support at a moment of need? | | X |
| Domain 4: The CDSS implementation | |  | |  |
| Information to users about the CDSS and its functions is appropriate | | - Is the communication and documentation about the CDS appropriate? | | X |
|  |  | - Are help topics related to the functioning of the CDS system available to users? | | X |
|  |  | - If necessary, is user training available? | | X |
| Other barriers and facilitators to compliance with the decision support advice are assessed/addressed | | - Is there an assessment of the beliefs, attitudes and skills of the healthcare providers and patients that may affect adherence to the decision support advice? Are actions planned/taken accordingly? | | X |
|  |  | - Is there an assessment of the professional interactions affecting adherence to the recommended actions, and are actions planned/ taken accordingly? | | X |
|  |  | - Is there an assessment of the (dis)incentives affecting the adherence of healthcare providers and patients to the decision support advice? Are necessary actions planned/taken? | | X |
|  |  | - Is there an assessment of the issues related to the capacity and resources needed to ensure adherence to the recommended actions? Are the necessary actions planned/taken? | | X |
|  |  | - Does the organisational context influence adherence to the decision support advice and what are actions planned/taken accordingly? | | X |
| Implementation is stepwise and the improvements in the CDSS are continuous | | - Is the implementation of the CDS stepwise? | |  |
|  |  | - Is a plan in place to collect user feedback and to monitor system usage, performance and outcomes? | |  |
|  |  | - Are malfunctions and other problems with use of the CDS quickly fixed? | |  |
| Governance of the CDSS implementation is appropriate | | - Are all the key stakeholders involved in the planning and implementation of the system? - Is the CDS initiative governed in an efficient, sustainable and equitable way? | |  |
