# Supplementary material for: Patient-Centered Clinical Decision Support for Recommended Screenings in French Primary Care: Protocol for a Multicenter Mixed Methods Feasibility Study
Source: JMIR Res Protoc. 2026 Sep 3;15:e89922. doi: 10.2196/89922 (PMC13586705; doi:10.2196/89922)
Supplement: Supplementary file 2 [file resprot_v15i1e89922_app2.docx]

**Multimedia Appendix 2:** SPIRIT 2013 checklist

| **Item** | **SPIRIT item (abridged)** | **Location in the manuscript** |
| --- | --- | --- |
| 1 | Title: descriptive, identifying design | Title page (design, population, and setting identified) |
| 2a-b | Trial registration; WHO dataset | Abstract and Results: ClinicalTrials.gov NCT07270926 |
| 3 | Protocol version (date, number) | NR |
| 4 | Funding sources | Results: CLARA, Métropole de Lyon, Région Auvergne-Rhône-Alpes (Proof of Concept 2023) |
| 5a | Roles and responsibilities of authors | Authors' Contributions |
| 5b-c | Sponsor contact and role | Funding statement |
| 5d | Committees (steering, coordination) | NR |
| 6a | Background and rationale | Introduction |
| 6b | Choice of comparators | N/A |
| 7 | Objectives | Study Objectives |
| 8 | Trial design | Study Design; Box 1, Figure 1 |
| 9 | Study setting | Setting |
| 10 | Eligibility criteria | Table 3 |
| 11a | Intervention description | The Lianeli CDSS; Timeline |
| 11b | Discontinuation/modification criteria | Table 3 (exclusion: consent withdrawal) |
| 11c | Adherence strategies | GP training; research assistant support for data collection |
| 11d | Concomitant care | Setting |
| 12 | Outcomes (incl. metric, method, time point) | Outcomes; Figure 1;Table 2 |
| 13 | Participant timeline | Table 2; Figure 1 |
| 14 | Sample size | Sample Size |
| 15 | Recruitment | Recruitment and Informed Consent |
| 16a-c / 17a-b | Allocation and blinding | N/A |
| 18a | Data collection methods | Data Collection and Management; Table 2 |
| 18b | Retention and follow-up | Data Collection and Management |
| 19 | Data management | Data Collection and Management (eCRF); Ethical Considerations (pseudonymization, storage) |
| 20a-c | Statistical methods; additional and missing-data analyses | Statistical Analysis |
| 21a-b | Data monitoring committee; interim analyses | Statistical Analysis |
| 22 | Harms | ⚠ Add one sentence on the (non-)collection of adverse events, consistent with the minimal-risk classification (RIPH category) of the study |
| 23 | Auditing | NR |
| 24 | Ethics approval | Ethical Considerations |
| 25 | Protocol amendments | Ethical considerations |
| 26a-b | Consent; ancillary use of data | Recruitment and Informed Consent; Ethical Considerations (CNIL authorization) |
| 27 | Confidentiality | Ethical Considerations |
| 28 | Declaration of interests | Conflicts of Interest |
| 29 | Access to data | Data Availability |
| 30 | Ancillary and post-trial care | N/A |
| 31a-c | Dissemination policy; authorship; public access to protocol | Results (publication planned 2027); Authors' Contributions |
| 32 | Informed consent materials | Ethical Considerations |
| 33 | Biological specimens | N/A |
