# Supplementary material for: Patient-Centered Clinical Decision Support for Recommended Screenings in French Primary Care: Protocol for a Multicenter Mixed Methods Feasibility Study
Source: JMIR Res Protoc. 2026 Sep 3;15:e89922. doi: 10.2196/89922 (PMC13586705; doi:10.2196/89922)
Supplement: Supplementary file 3 [file resprot_v15i1e89922_app3.docx]

**Multimedia Appendix 3:** Patient Guideline Implementation with Decision Support (GUIDES) questionnaire.

| **GUIDES criterion** | **Patient-reported item** | **Variable format** |
| --- | --- | --- |
| Perceived relevance of the intervention | I have a better understanding of the screenings recommended for me | 7-point Likert scale* + “Not applicable” option |
| Perceived relevance of the intervention | The information provided helped me decide |  |
| Perceptions/attitude toward the intervention | My experience using Lianeli was positive |  |
| Perceptions/attitude toward the intervention | In the future, I would agree to use Lianeli with my physician |  |
| Trust in the provided information | I trust the list of screenings recommended for me |  |
| Trust in the provided information | I trust the screening information sheets |  |
| Relevance of the provided information | The information provided (by Lianeli and/or by your physician) answered my questions |  |
| Perceived clinical importance | I have other health priorities |  |
| Information overload | I received too much information |  |
| CDSS usability | F-SUS questionnaire, 10 items[17] |  |
| Information format | The visual format of the information presented in Lianeli was satisfactory |  |
| Information format | The information presented in Lianeli was understandable |  |
| Timing of information delivery | I found it acceptable to learn the list of recommended screenings on Lianeli (before seeing my physician) |  |

* 7-point Likert anchors: (1) Strongly disagree; (2) Disagree; (3) Somewhat disagree; (4) Neither agree nor disagree; (5) Somewhat agree; (6) Agree; (7) Strongly agree
