# Supplementary material for: Patient-Centered Clinical Decision Support for Recommended Screenings in French Primary Care: Protocol for a Multicenter Mixed Methods Feasibility Study
Source: JMIR Res Protoc. 2026 Sep 3;15:e89922. doi: 10.2196/89922 (PMC13586705; doi:10.2196/89922)
Supplement: Supplementary file 4 [file resprot_v15i1e89922_app4.docx]

**Multimedia Appendix 4:** Primary care professional (PCP) Guideline Implementation with Decision Support (GUIDES) questionnaire.

| **GUIDES criterion** | **Measured variable (HCP-reported item)** | **Variable format** |
| --- | --- | --- |
| Perceived relevance of the intervention | The intervention facilitates identifying my patients’ eligibility for recommended screenings | 7-point Likert scale* + “Not applicable” option |
| Perceived relevance of the intervention | The intervention facilitates shared decision-making |  |
| Affective attitude | My experience using Lianeli was positive |  |
| Workload burden | I find it feasible to integrate Lianeli into my workflow |  |
| Trust in the provided information | I trust the information provided by Lianeli |  |
| Relevance of the provided information | I find the information provided by Lianeli relevant |  |
| Perceived clinical importance | I have other priorities for my patients’ health |  |
| Information overload | I experience information overload when using Lianeli |  |
| Lianeli usability | F-SUS questionnaire 10 items[17] |  |
| Information format | The visual format of the information presented in Lianeli was satisfactory |  |
| Timing of information delivery | I appreciated receiving the list of screenings for which my patient is eligible before the consultation |  |
| Training to use Lianeli | My training to use Lianeli was of good quality |  |

* 7-point Likert anchors: (1) Strongly disagree; (2) Disagree; (3) Somewhat disagree; (4) Neither agree nor disagree; (5) Somewhat agree; (6) Agree; (7) Strongly agree.
