# Supplementary material for: Patient-Centered Clinical Decision Support for Recommended Screenings in French Primary Care: Protocol for a Multicenter Mixed Methods Feasibility Study
Source: JMIR Res Protoc. 2026 Sep 3;15:e89922. doi: 10.2196/89922 (PMC13586705; doi:10.2196/89922)
Supplement: Supplementary file 5 [file resprot_v15i1e89922_app5.docx]

**Multimedia Appendix 5:** Predefined qualitative canvas, according to the Guideline Implementation with Decision Support (GUIDES) framework.

| **THEMES** | **Other PCPs** | **GP** | **Patient** |
| --- | --- | --- | --- |
| Perceived relevance of the ADER-F intervention (difficulty identifying screening eligibility; difficulty accessing information on benefits and harms) | X | X | X |
| Determinants of overall attitude toward ADER-F (positive/negative: why) | X | X | X |
| Data quality: does it need improvement? If yes, how? | X | X |  |
| Strengths and weaknesses of ADER-F regarding impact on GPs’ workload |  | X |  |
| Perceived importance of the information presented for patients and GPs; satisfaction with how Lianeli presents levels of evidence |  | X | X |
| Strengths and weaknesses of ADER-F for shared decision-making |  | X | X |
| Missing information in Lianeli and how it should be presented | X | X | X |
| Is it useful to ask patients what they want to know before offering information sheets? (information needs) |  |  | X |
| Importance of algorithm explainability: why? Are Lianeli’s outputs sufficiently explainable? |  | X | X |
| Perceived clinical value of a screening-dedicated consultation |  | X | X |
| Feasibility of implementing a screening-dedicated consultation (workload burden) |  | X | X |
| Barriers and facilitators related to usability of the CDSS | X | X | X |
| Barriers and facilitators related to the format of delivered information |  | X | X |
| Interests, barriers and facilitators related to interprofessional collaboration with Lianeli | X | X | X |
| Views on the timing of information delivery: (1) eligibility for screenings, (2) benefits/harms of screenings | X | X | X |
| Concerns about data security, conflicts of interest, and ethical/regulatory issues | X | X | X |
